# Teaching module for obesity bias education: incorporating comprehensive competencies and innovative techniques

**DOI:** 10.1186/s12909-023-04310-4

**Published:** 2023-05-16

**Authors:** Jessica Koran-Scholl, Jenenne Geske, Karl R. Khandalavala, Birgit Khandalavala

**Affiliations:** 1 Department of Family Medicine, University of Nebraska Medical Center, 983075 Nebraska Medical Center, Omaha, NE 68198-3075 USA; 2 Mayo Clinic College of Medicine and Science, 200 First ST SW, 55905 Rochester, MN USA

**Keywords:** Obesity Bias, Explicit, Implicit, E-Learning, Resident Education

## Abstract

**Background:**

The majority of the United States population is overweight or obese, and obesity bias is frequently reported by patients. Obesity bias is associated with adverse health outcomes, even independent of body weight. Primary care residents are often sources of obesity bias towards patients with weight, yet education regarding obesity bias is significantly lacking in most family medicine residency teaching curricula. The aim of this study is to describe an innovative web-based module on obesity bias and discuss its impact in family medicine residents.

**Methods:**

The e-module was developed by an interprofessional team of health care students and faculty. It consisted of a 15-minute video containing five clinical vignettes that depicted instances of explicit and implicit obesity bias in a patient-centered medical home (PCMH) model. Family medicine residents viewed the e-module as part of a dedicated one-hour didactic on obesity bias. Surveys were administered prior to and following the viewing of the e-module. They assessed previous education on obesity care, comfort in working with patients with obesity, residents’ understanding of their own biases in working with this population, and the anticipated impact of the module on future patient care.

**Results:**

A total of 83 residents from three family medicine residency programs viewed the e-module and 56 completed both the pre and post survey. There was a significant improvement in residents’ comfort in working with patients with obesity as well as their understanding of their own biases.

**Conclusion:**

This teaching e-module is a short, interactive, web-based educational intervention that is free and open-sourced. The first-person patient perspective allows learners to better understand the patient’s point of view and its PCMH setting illustrates interactions with a variety of healthcare professionals. It was engaging and well received by family medicine residents. This module can begin the conversation around obesity bias, leading to improved patient care.

**Supplementary Information:**

The online version contains supplementary material available at 10.1186/s12909-023-04310-4.

## Introduction

Since the 1990s, obesity rates have rapidly increased from under 10% to a current prevalence of 41.9% in the United States (US) [1]. Over the past decade, obesity consistently ranked as the leading cause of morbidity and mortality in the US. Consequently, obesity is emerging as the most prominent public health issue of the twenty-first century. In most US states, estimates of obesity prevalence are projected to be above 50% by the year 2030 [1, 2]. For women, minorities (non-Hispanic black adults), low-income, and other vulnerable populations, prevalence rates may be much higher, with projections for obesity prevalence peaking at the intersections of these groups, potentially exacerbating preexisting poor health outcomes and treatments [3, 4].

The most recent studies estimate that 42–61% of US adults have experienced weight bias with effects that may be independent of body weight [5, 6]. Many studies clearly demonstrate widespread bias among medical providers and medical students towards patients of size [7–9]. Obesity bias can be explicit, manifested overtly by the medical provider towards the patient, or implicit, more subtle or subconscious biased provider interactions. While rates of obesity bias in the US are comparable in prevalence to those of racial bias, to date, the effects of obesity bias are largely underrecognized in medical practice [10, 11].

Patients experiencing weight bias may avoid preventative and other medical care and a vicious cycle may be initiated, resulting in an increased risk of new medical conditions or aggravation of existing diagnoses [12]. Mental health is also commonly impacted, with increased psychological distress, including substance use and suicidal ideation, in patients who perceive weight bias [13, 14]. Higher rates of mortality were noted in patients with obesity stigma that could not be accounted for by common physical and psychological risk factors. The association between mortality and weight bias was noted to “be generally stronger than that between mortality and other attributions for discrimination” and “may shorten life expectancy.” [15].

Since 2007, the Association of American Medical Colleges (AAMC) has recommended that the medical education of future physicians incorporate respectful and effective care of patients with overweight and obesity [16, 17]. In recent years, several organizations have worked to develop standards and competencies for addressing care to patients with obesity and to mitigate obesity bias [18, 19]. In 2017, the Provider Training and Education Workgroup of the Integrated Clinical and Social Systems for the Prevention and Management of Obesity Innovation Collaborative detailed ten Provider Competencies for the Prevention and Management of Obesity. One of the competencies and its associated sub-competencies specifically address obesity bias [18].

7. Employ strategies to minimize bias towards and discrimination against people with obesity, including weight, body habitus, and the causes of obesity.
7.1 Describe the ways in which weight bias and stigma impact health and
  wellbeing
7.2 Recognize and mitigate personal biases.
7.3 Recognize and mitigate the weight biases of others.

These guidelines reinforce the need for clinically focused education to mitigate effects of obesity bias in the primary care setting.

Recommendations for teaching obesity bias, include educating learners about the complexities and biological processes involved in weight issues, as well as removing personal blame for weight gain [10, 20]. It has long been noted that there is a deficit in obesity education in primary care residencies, and it has been challenging to implement obesity medicine curricula to address these issues [21, 22]. An already crowded didactic schedule, limited faculty knowledge about obesity, and negative attitudes about obesity as a disease have been highlighted as potential barriers to implementation of obesity curricula [17].

There is a paucity of validated teaching material for obesity bias, especially regarding implicit biases, and few programs have implemented any instruction around the subject of obesity bias [20, 23]. Efficient and effective educational materials for students and residents could help overcome educational deficiencies [17, 22].

E-Learning and distance learning are well established and extensively documented [24]. However, integrating electronic resources, which can take the form of videos, questions, or interactive web-based modules (e-modules), into a structured curriculum is distinct from distance learning [25]. While e-modules offer flexibility in self-directed learning, to date there is no report of this methodology being used for obesity bias instruction for medical students and residents. The aim of this present study is to describe an innovative web-based module (e-module) on obesity bias. In addition, we discuss its impact in family medicine residents on bias awareness and perceived comfort in their care for patients with obesity.

## Methods

An interprofessional team of health care students (2 medical, 1 physician assistant, 1 pharmacy student, 1 social work, and 1 public health) developed a 15-minute interactive web-based e-module on obesity bias under the oversight of obesity medicine experts and a clinical psychologist. Content was based on the Provider Competency guidelines mentioned above, personal experiences of the creators, and the UConn Rudd center [18, 24]. The finished e-module was presented as part of a university-wide e-Learning showcase.

The interactive module depicts five clinical vignettes depicting problematic patient/physician contact in a non-judgmental setting. The vignettes are presented from a first-person patient perspective, intentionally refraining from providing any direct visualization of the patient. This more easily allows learners to experience the patient’s point of view as the visit unfolds.

The vignettes occur in a Patient-Centered Medical Home (PCMH) setting and include video clips depicting the patient’s clinical interactions with a medical assistant, physician, dietitian, pharmacist, and social worker (Table 1). They incorporate both implicit and explicit aspects of obesity bias, with clear examples and questions, enabling learners to recognize and label distinct instances of bias. For instance, implicit bias was noted in the e-module when the physician had poor eye contact and lack of empathy with the patient. Explicit bias was noted in the e-module when the physician suggested the patient should “push back” from the table more often. The e-module reinforces the learning objectives, concluding with a 10-question knowledge assessment addressing the causes of obesity, types of obesity bias, and recognition of bias and mitigation strategies.

**Table 1:** E-Learning module components and content focus as encountered in a Patient Centered Medical Home (PCMH) setting

Patient-Centered Medical Home Encounters	Content focus
Medical Assistant	Obtaining vitals/blood pressure
Physician	Contribution of weight to chronic medical problems
Dietitian	Avoiding junk food and making healthy choices
Pharmacist	Medication for co-morbid diabetes and weight loss
Social Worker	Obtaining healthy food in the community

Since lack of time is a well-known limiting factor in medical education, this educational resource was produced and edited with a focus on brevity, with a total length of 15 min, including the self-assessment summary. This is a free, closed-captioned, and open-sourced module that anyone can access to enhance their patient-centered communication skills and is available on the university e-gallery website (https://www.unmc.edu/elearning/egallery/obesity-bias/).

Residents from three family medicine residency programs completed the e-module independently and prior to attending a one hour in-person didactic on the biopsychosocial management of obesity in primary care. Specifically, the didactic included education regarding the biological complexity of obesity along with obesity bias and its mitigation strategies. Residents were sent an electronic link to the pre-survey, which generated a link to the e-module upon completion. The pre-survey assessed previous education on obesity, their level of comfort in working with patients with obesity and their understanding of their own biases in working with this population. The post-survey was administered immediately following the didactic and also assessed their level of comfort in working with patients with obesity and their understanding of their own biases along with their estimate of the impact of the module on their future patient care. Questions addressing their comfort and understanding of their biases included a 4-point scale, where 1 = Not at all and 4 = Mostly. The post-survey also included open-ended qualitative questions evaluating the module itself. Institutional Review Board (IRB) approval was obtained (574-16-EX).

### Data analysis

Descriptive statistics were used to summarize the quantitative data, and paired sample t-tests examined the change from pre to post on their comfort and understanding of their biases. Quantitative data analysis was done using SPSS v27.0. Qualitative responses were analyzed separately by each member of the research team using an immersion/crystallization process. The team members then collectively agreed on common overarching themes.

## Results

A total of 83 residents completed the e-module. Of these, 73 (88%) completed the pre-survey, and 66 (80%) completed the post-survey; 56 (67%) participants completed both. The educational initiative was mandatory for all residents. However, the completion of the pre/post surveys was voluntary. The ten knowledge assessment questions that were part of the e-module were for the learners benefit only and responses could not be retained. In the pre-survey, 64% of residents reported no prior focused education about the biopsychosocial management of obesity in their medical school curriculum. Results of a paired sample t-test, including only those 56 participants who completed both the pre- and post-surveys, indicate that comfort level increased significantly after completing the module (p = 0.001). The mean level of comfort in working with patients with obesity increased from a mean of 3.0 (on a 4.0 scale) (SD = 0.7) to a mean of 3.30 (SD = 0.6; p = 0.001) (Table 2).

**Table 2:** Survey questions, before and after module completion

**Have you received any education about the biopsychosocial management of obesity?**										
	**No**	**Yes**								
**Pre**	47 (64.4%)	26 (34.6%)								
**How comfortable do you feel working with patients with obesity?** ^a^										
	**Not at all**	**Slightly**	**Moderately**		**Mostly**		**Mean**		**p-value** ^b^	
**Pre**	2 (2.7%)	12 (16.4%)	41 (56.2%)		18 (24.7%)		3.00		0.001	
**Post**	0 (0.0%)	5 (7.6%)	37 (56.1%)		24 (36.4%)		3.30			
**How well do you think you understand your biases in working with patients with obesity?** ^a^										
	**Not at all**	**Slightly**		**Moderately**	**Mostly**		**Mean**			**p-value** ^b^
**Pre**	2 (2.7%)	24 (32.9%)		37 (50.7%)	10 (13.7%)		2.77			< 0.001
**Post**	0 (0.0%)	2 (3.0%)		31 (47.0%)	33(50.0%)		3.48			
**To what degree will what you learned today impact your approach to working with patients with obesity?**										
	**Not at all**	**Slightly**		**Moderately**	**Mostly**					
**Post**	1 (1.2%)	2 (2.4%)		25 (30.1%)	38 (45.8%)					

^a^ Scored on a scale where Not at all = 1, Slightly = 2, Moderately = 3, and Mostly = 4

^b^ Based on a Wilcoxon Signed Rank test

Similarly, when respondents were asked how well they understood their own biases in working with patients with obesity, scores increased from a mean of 2.77 (on a 4.0 scale) (SD = 0.7) to a mean of 3.48 (SD = 0.5; p < 0.001). Residents overwhelmingly endorsed the module; 95.5% percent of residents said that what they learned would moderately or mostly impact their approach to working with patients with obesity.

Qualitative analysis revealed that residents found the e-module engaging and enabled them to reflect on the comprehensive nature of caring for patients with obesity (Table 3).

**Table 3:** Qualitative themes emerging from e-learning module viewers’ participant feedback

**What did you enjoy about the e-module?**
1. **Engaging** • “Simple, interactive” • “Easy, friendly direct and funny” • “I thought that the patient interactions were entertaining and informative which makes them very useful. Too many e-module are too long. Well done!” • “It was informative and engaging”
2. **Presents Patient Perspective** • “Showed how much body language and non-verbal cues can affect patients’ visit” • “I enjoyed having the first-person perspective about how patients struggle with their weight feel and perceive their experiences in healthcare. I think seeing the interactions through the patients’ eyes helps highlight things that healthcare workers may not notice from their perspective.” • “The interactive videos help show how the things we say /do have an impact on our patients’ experiences in the healthcare setting.”
3. **Authentic Illustration of a Patient Centered Medical Home (PCMH) visit** • “Helps point out bias in real situations” • “I enjoyed the authenticity of the e-learning module and how real it felt.” • “Raised awareness about challenging patients with obesity have in a clinical setting” • “The videos provided excellent examples of bias in the clinical setting” • “Realistic interactions” • “I enjoyed that it was very representative of real-world scenarios”
4. **Reflective** • “Very important topic especially for our outpatient population. Great to address and self-reflect on biases and how we present obesity related topics with our patients” • “Very nice examples of implicit bias. Will help me address obesity with my clinic patients” • “We all have biases that we need to be aware of.” • “Used situations and statements by providers that seem like no one would say, but it makes a point that with reflection I know I have directly seen in clinic. Makes me reflect on interactions I have seen in clinic and my own interactions.”
**What areas could be improved in the e-module?**
1. **Provide mitigation strategies** • “Include opportunities to learn strategies to overcome bias and improve patient care for patients with obesity” • “Create video examples of what a positive patient experience looks like strategies” • “I totally feel they don’t fully encapsulate the realities that providers may face when treating patients of any size that are chronically obese with little to no healthcare compliance i.e patient who says they have good blood sugar despite having an A1C of 10 and a glucose > 200” • “Now that we are more aware of implicit bias & explicit bias, what resources are there to address the patient with obesity-include in E-module” • “While it is critical to improve how we address obesity with our patients, there still are many instances when weight must be addressed to help our patients address their health. Would be helpful to provide more tips for these conversations when it is not biased, but good medical care, to help patients lose weight”
2. **Increase Difficulty** • “More questions to dig deeper into bias” • “While humorous, some of the dialogue was hyperbolic could be improved through making the ‘biased statements’" reflect subtle examples of bias.”

Responses indicated that residents enjoyed seeing an interaction from a patient’s perspective and that they appreciated the authenticity of an interprofessional PCMH model of caring for patients with obesity. Residents suggested that the module could be improved by including more strategies to mitigate obesity bias in patient encounters and making some of the examples more complex, thereby increasing the difficulty.

## Discussion

We describe an innovative video module that illustrates how obesity bias might be perceived from the point of view of a patient with obesity in a clinical setting. This e-module incorporates Provider Competencies specifically addressing obesity bias, enabling providers and trainees to better recognize and mitigate obesity bias. Family medicine residents using this interactive e-module reported significantly increased awareness of personal biases regarding obesity, increased comfort treating patients with obesity, and favorable ratings of the clinical applicability of the e-module.

To date, there are very few studies on techniques for addressing obesity bias [20]. Recently a seminal framework has been developed to guide educators in teaching implicit bias [26]. Additional papers have included methods for increasing awareness of obesity bias [27]. One study attempted to utilize faculty / resident role modeling to increase positive contact between students and patients with obesity while eliminating unprofessional behaviors [28]. Several studies have incorporated both favorable and unfavorable representations of physician/patient interactions to address obesity bias, including case-based discussions [29] play reading, [30] visiting an art museum, [31] and ethics discussions after watching scenes from a TV medical drama [32].

These different methods primarily take a third-person perspective in each clinical scenario. In contrast, the e-module described in the present study utilizes first-person patient perspective. Although potential gender bias from voice recordings of the patient could not be eliminated, use of the first-person perspective eliminated concomitant race or age bias by avoiding direct visualization of the patient. This perspective more easily allows learners to experience the patient’s point of view as the visit unfolds and was repeatedly referenced as effective in resident surveys in this study.

The UConn Rudd Center for Food Policy and Health produced a 17-minute video depicting a single visit between a patient with obesity and their provider [24]. Our video, while similar in some respects, has some differences. Our content was created by learners from a variety of professional backgrounds and body sizes, to ensure learner input and size inclusivity. The “patient” in our video is not portrayed in any scene, and the encounters are depicted as they would be seen by the patient. The PCMH setting included a wide variety of healthcare professionals and addressed instances of implicit and explicit bias. Our module allowed interaction by the learner and included reflection questions designed to align with the Provider Competencies for the Prevention and Management of Obesity. Interactive learning modules have been used for teaching health care trainees [33]. We noted that the vignettes prompted discussion while helping learners build insight and greater awareness of how obesity bias can occur during patient encounters.

This e-module can be fully incorporated into a completely remote delivery as a stand-alone educational tool or in conjunction with a more robust obesity education curriculum. Because it is open-sourced, it can be accessed by anyone wishing to strengthen their communication skills, particularly when interacting with patients of size.

Limitations of our study consist of lack of precedence of similar methodology and exceedingly limited validated resources and published studies [17, 20]. Another limitation is the relatively small sample, although we have continued to incorporate the module into our obesity teaching sessions and are in the process of collecting further data. Additionally, our pre/post survey was limited in scope. Additional research is needed to determine how our e-module impacts objective measures of performance in patient care. We were unable to find validated instruments assessing obesity bias in healthcare providers, and we wanted to keep our survey simple and short. As more robust questionnaires are validated to measure change in implicit and explicit bias, we will incorporate these questions into future studies.

## Conclusion

Graduate and undergraduate teaching programs have limited resources and have a low priority in teaching obesity and obesity bias. Our study indicates that a brief web-based interactive video e-module can serve as an engaging and stimulating methodology to begin the conversation around obesity bias using patient-centric clinical vignettes. Given the projected obesity rates in the US, we advocate for medical educators to be aware of the critical need for development and dissemination of patient-centered educational material to optimize the care of our patient population.

## Electronic supplementary material

Below is the link to the electronic supplementary material.

Supplementary Material 1

## Data Availability

The dataset generated and/or analyzed during the current study is available in the Harvard Dataverse repository and can be accessed at 10.7910/DVN/OXSXR0.
