# Supplementary material for: Teaching module for obesity bias education: incorporating comprehensive competencies and innovative techniques
Source: BMC Med Educ. 2023 May 16;23:340. doi: 10.1186/s12909-023-04310-4 (PMC10190021; doi:10.1186/s12909-023-04310-4)
Supplement: Supplementary file 1 — Supplementary Material 1 [file 12909_2023_4310_MOESM1_ESM.pdf]

MEDIA AUTHORIZATION FORM

In accordance with the Family Educational Rights and Privacy Act (FERPA), the undersigned student hereby grants to the Board of Regents of the University of Nebraska, its campuses, its representatives, employees, agents and assigns, the irrevocable and unrestricted right to use, reproduce and publish photo/video of me, including my image and likeness as depicted therein, which are identified below, for editorial, trade, advertising or any other purpose and in any manner and medium, and to alter the same without restriction and to copyright the same.

I hereby release the University of Nebraska, its components, campuses and its Regents, officers, employees, agents, legal representatives and assigns from any and all claims, actions and liability related to its use of said photographs.

I recognize by signing below, I waive any right to royalties or other compensation arising from or related to the use of the photo and/or video. My signature below also grants the University of Nebraska the use of my image for seven years from the date on this form.

UNMC E-Learning Program

(Name of event, project, and/or location)

11/8/18

Date

[signature]

Student Signature or parent/guardian if age 18 or under

STUDENT INFORMATION:

402-203-9989

Phone

Karl Khandalavala

Printed Name

Omaha, NE 68124

Current AND Permanent Address (City, State, Zip)

Karl.Khandalavala@unmc.edu

Email Address

REQUESTOR:	Other information:
Peggy Moore, E-Learning, Director	
Name/Department/Title	
peggy.moore@unmc.edu, 402.559.8082	
Contact Information	

UNMC
BREAKTHROUGHS FOR LIFE.®

MEDIA AUTHORIZATION FORM

In accordance with the Family Educational Rights and Privacy Act (FERPA), the undersigned student hereby grants to the Board of Regents of the University of Nebraska, its campuses, its representatives, employees, agents and assigns, the irrevocable and unrestricted right to use, reproduce and publish photo/video of me, including my image and likeness as depicted therein, which are identified below, for editorial, trade, advertising or any other purpose and in any manner and medium, and to alter the same without restriction and to copyright the same.

I hereby release the University of Nebraska, its components, campuses and its Regents, officers, employees, agents, legal representatives and assigns from any and all claims, actions and liability related to its use of said photographs.

I recognize by signing below, I waive any right to royalties or other compensation arising from or related to the use of the photo and/or video. My signature below also grants the University of Nebraska the use of my image for seven years from the date on this form.

UNMC E-Learning Program
(Name of event, project, and/or location)

11/8/18 — Date

Ben Fletcher (signature) — Student Signature or parent/guardian if age 18 or under

STUDENT INFORMATION:

(402) 779-5416 — Phone

Ben Fletcher — Printed Name

9950 Garvin St. Omaha, NE 68122
Current AND Permanent Address (City, State, Zip)

ben.fletcher@unmc.edu
Email Address

REQUESTOR:	Other information:
Peggy Moore, E-Learning, Director<br>Name/Department/Title	
peggy.moore@unmc.edu, 402.559.8082<br>Contact Information	

University of Nebraska Medical Center

MEDIA AUTHORIZATION FORM

UNMC
BREAKTHROUGHS FOR LIFE.™

In accordance with the Family Educational Rights and Privacy Act (FERPA), the undersigned student hereby grants to the Board of Regents of the University of Nebraska, its campuses, its representatives, employees, agents and assigns, the irrevocable and unrestricted right to use, reproduce and publish photo/video of me, including my image and likeness as depicted therein, which are identified below, for editorial, trade, advertising or any other purpose and in any manner and medium, and to alter the same without restriction and to copyright the same.

I hereby release the University of Nebraska, its components, campuses and its Regents, officers, employees, agents, legal representatives and assigns from any and all claims, actions and liability related to its use of said photographs.

I recognize by signing below, I waive any right to royalties or other compensation arising from or related to the use of the photo and/or video. My signature below also grants the University of Nebraska the use of my image for seven years from the date on this form.

UNMC E-Learning Program
(Name of event, project, and/or location)

11/8/18 — Date

Brooke Fletcher — Student Signature or parent/guardian if age 18 or under

STUDENT INFORMATION:

402-689-9694 — Phone

Brooke Fletcher — Printed Name

7003 Western Ave Omaha NE 68132 — Current AND Permanent Address (City, State, Zip)

Permanent: 9950 Garvin Rd Omaha, NE 68122

brooke.fletcher@unmc.edu — Email Address

REQUESTOR:	Other information:
Peggy Moore, E-Learning, Director<br>Name/Department/Title<br><br>peggy.moore@unmc.edu, 402.559.8082<br>Contact Information	

University of Nebraska Medical Center

UNMC
BREAKTHROUGHS FOR LIFE.

MEDIA AUTHORIZATION FORM

In accordance with the Family Educational Rights and Privacy Act (FERPA), the undersigned student hereby grants to the Board of Regents of the University of Nebraska, its campuses, its representatives, employees, agents and assigns, the irrevocable and unrestricted right to use, reproduce and publish photo/video of me, including my image and likeness as depicted therein, which are identified below, for editorial, trade, advertising or any other purpose and in any manner and medium, and to alter the same without restriction and to copyright the same.

I hereby release the University of Nebraska, its components, campuses and its Regents, officers, employees, agents, legal representatives and assigns from any and all claims, actions and liability related to its use of said photographs.

I recognize by signing below, I waive any right to royalties or other compensation arising from or related to the use of the photo and/or video. My signature below also grants the University of Nebraska the use of my image for seven years from the date on this form.

Obesity Bias E-Learning Module
(Name of event, project, and/or location)

11/20/18 [signature]
Date — Student Signature or parent/guardian if age 18 or under

STUDENT INFORMATION:

612-210-9588 — Anthony Marchio
Phone — Printed Name

3804 Farnam St. Apt. 309 Omaha, NE, 68131
Current AND Permanent Address (City, State, Zip)

anthony.marchio@unmc.edu
Email Address

REQUESTOR:	Other information:
Name/Department/Title	
Contact Information	

University of Nebraska Medical Center
